# Error and Bias in Determining Exposure Potential of Children at School Locations Using Proximity-Based GIS Techniques

**DOI:** 10.1289/ehp.9668

**Published:** 2007-05-15

**Authors:** Paul A. Zandbergen, Joseph W. Green

**Affiliations:** 1 Department of Geography, University of New Mexico, Albuquerque, New Mexico, USA; 2 Department of Geography, University of South Florida, Tampa, Florida, USA

**Keywords:** bias, children, data quality, error sources, geocoding, geographic information systems (GIS), positional accuracy, schools, vehicle emissions

## Abstract

**Background:**

The widespread availability of powerful tools in commercial geographic information system (GIS) software has made address geocoding a widely employed technique in spatial epidemiologic studies.

**Objective:**

The objective of this study was to determine the effect of the positional error in geocoding on the analysis of exposure to traffic-related air pollution of children at school locations.

**Methods:**

For a case study of Orange County, Florida, we determined the positional error of geocoding of school locations through comparisons with a parcel database and digital orthophotography. We used four different geocoding techniques for comparison to establish the repeatability of geocoding, and an analysis of proximity to major roads to determine bias and error in environmental exposure assessment.

**Results:**

Results indicate that the positional error in geocoding of schools is very substantial: We found that the 95% root mean square error was 196 m using street centerlines, 306 m using TIGER roads, and 210 and 235 m for two commercial geocoding firms. We found bias and error in proximity analysis to major roads to be unacceptably large at distances of < 500 m. Bias and error are introduced by lack of positional accuracy and lack of repeatability of geocoding of school locations.

**Conclusions:**

These results suggest that typical geocoding is insufficient for fine-scale analysis of school locations and more accurate alternatives need to be considered.

Advances in geographic information systems (GIS), statistical methodology, and availability of high-resolution georeferenced health and environmental data have created unprecedented opportunities for spatial epidemiology to investigate local geographic variation in disease (Elliot and Wartenberg 2004). GIS has become widely used to locate the study population by geocoding addresses, using proximity analysis of pollution sources as a surrogate for exposure, and integrating environmental monitoring data into the analysis of health outcomes (Nuckols et al. 2004). As the capabilities of GIS have improved, address geocoding has become a very accessible research methodology, and as a result the individual address is becoming a standard level of spatial investigation. Geocoding can introduce bias and error (Rushton et al. 2006) but the effect this has on the results of epidemiologic studies has received limited attention. In this study we explored the effect of positional error in geocoding using a case study of the exposure potential of children at school locations to traffic-related air pollution.

There are many potential problems with geocoding, which have been well described in the literature (Harries 1999; Krieger et al. 2001; Ratcliffe 2001; Rushton et al. 2006; Whitsel et al. 2004). Research on the quality of geocoding has emphasized a consideration of completeness, positional accuracy, and repeatability (Whitsel et al. 2004). The potential bias and error introduced by variability in match rates has received most attention (Hurley et al. 2003; Oliver et al. 2005). The effects of the positional accuracy and repeatability of geocoding has received limited attention and is therefore the subject of this study.

Several studies have determined quantitative estimates of the positional accuracy of geocoding. Estimates of typical positional errors range from 38 to 75 m (Bonner et al. 2003; Cayo and Talbot 2003; Dearwent et al. 2001; Karimi and Durcik 2004; Ratcliffe 2001; Ward et al. 2005) based on mean or median values. Results in urban areas are generally more accurate than in rural areas (Bonner et al. 2003; Cayo and Talbot 2003; Ward et al. 2005). This suggests that the positional error of geocoding can be substantial and needs to be characterized in a meaningful manner relevant to the use of the geocoded locations. Particularly with reference to epidemiologic studies, when short distances are associated with health effects, the geocoding result must have a positional accuracy that is sufficient to resolve whether such effects are present (Rushton et al. 2006).

Vehicular traffic–related emissions are a major source of air pollution, especially in urban areas. Proximity to busy roads has been associated with health effects in children, particularly respiratory symptoms and asthma (Brauer et al. 2002; Brunekreef et al. 1997; Ciccone et al. 1998; Edwards et al. 1994; Gauderman et al. 2005; Janssen et al. 2001; Kim et al. 2004; Lewis et al. 2005; Morris et al. 2000; Nicolai et al. 2003; van Vliet et al. 1997; Venn et al. 2000, 2001; Zmirnou et al. 2004). Several studies have also found associations between proximity to traffic and higher rates of childhood cancer (Pearson et al. 2000; Raaschou-Nielsen et al. 2001; Savitz and Feingold 1989), but not all studies have been conclusive in this regard (Langholz et al. 2002; Reynolds et al. 2002).

Children were chosen as the subject of our study because they represent the largest portion of the population that is susceptible to environmental health risks, and air pollution in particular (Kim 2004; Schwartz 2004). The selection of school locations reflects a longstanding interest to consider time–activity patterns in exposure assessment (Duan 1982; Sexton and Ryan 1998). Many factors affect the exact nature of time–activity patterns (McCurdy and Graham 2003), but several studies confirm that for children schools represent the second most important location (after the home) to consider in environmental exposure analysis (Klepeis et al. 2001; Leech et al. 2002; Schwab et al. 1992; Xue et al. 2004).

Many studies have documented that the concentration of traffic pollution drops off rapidly with increasing distance from the road (Briggs et al. 2000; Gilbert et al. 2003, 2005; Hitchins et al. 2000; Kuhler et al. 1988; Morawska et al. 1999; Ross et al. 2006; Van Vliet et al. 1997; Venn et al. 2001; Wrobel et al. 2000; Zhu et al. 2002a, 2002b). Concentrations are highest near roadways, decrease rapidly after an exponential function, and reach near-background levels at approximately 300–500 m from the road. Based on this strong spatial gradient in pollutant concentrations, measuring proximity to major roads using GIS has become a widely employed alternative to actual exposure monitoring. In a typical analysis scenario, one or more buffer sizes are used to determine whether geocoded locations fall within certain distances from major roads. Most studies use only a single buffer distance, including 100 m (Giordian et al. 2006), 150 m (Gauderman et al. 2005; Green et al. 2004), 169 m (English et al. 1999), 229 m (Wilhelm and Ritz 2003), 300 m (Zmirnou et al. 2004), and 457 m (Langholz et al. 2002). Several other studies have used multiple distances ranging from 30 to 300 m (Hoek et al. 2002; Lewis et al. 2005; McConnell et al. 2006; Ong et al. 2006). Although the use of discrete buffer distances has been criticized for not capturing the true distance–exposure relationship (Maantay 2002; Zandbergen and Chakraborty 2006), their use is justified by the strong empirical evidence that pollutant concentrations follow a relatively predictable and rapid decrease with distance.

Studies on the effect of traffic-related air pollution have also considered traffic volume in the determination of environmental exposure conditions; adverse effects are observed for traffic counts starting at about 25,000 vehicles per day (Edwards et al.1994; English et al. 1999; Wijst et al. 1993). This value has become the lower exposure threshold used in studies that have modeled the potential exposure based on traffic counts and proximity (Green et al. 2004; Houston et al. 2006; Ong et al. 2006).

Various proximity-based metrics have been employed to relate traffic counts to exposure, including distance to the nearest major roadway with a high traffic count per day (Gauderman et al. 2005; Green et al. 2004; Lewis et al. 2005), the sum of traffic count within a buffer (Ong et al. 2006), distance-weighted traffic density (English et al., 1999; Gauderman et al. 2005; Pearson et al. 2000; Wilhelm and Ritz, 2003; Zmirnou et al. 2004), and traffic count at the nearest road (Raaschou-Nielsen et al. 2001). Studies comparing these traffic metrics to actual exposure to traffic-related pollutants have been few (Briggs et al. 2000; Gauderman et al. 2005), but they suggest pollutant concentrations correlate with distance from nearest road, traffic counts, and modeled air pollution. The distance to major road metric has been suggested as a reasonable, relatively easy-to-visualize metric for descriptive purposes (Green et al. 2004). Proximity to major roads is also computationally easy to estimate from data that are readily available compared with the meteorologic and traffic volume data required to model exposure conditions.

Several types of measurement error exist in exposure assessment to traffic-related air pollution (Van Atten et al. 2005). For example, Molitor et al. (2006) documented the effect of missing exposure data at the individual level and determined that the methodology chosen to account for this missing data influences the conclusions regarding the observed health effects. Another type of measurement error is the positional error in the various spatial data sets used to derive exposure estimates, which is the focus of this study.

Given that most studies on the exposure of children to air pollution from traffic have used relatively short distances of 50–500 m to major roadways with traffic counts of ≥ 25,000 vehicles, the question arises whether the geocoded locations of schools are accurate enough to allow for this type of proximity analysis. Several types of positional errors can be identified, including error in the major road network used for vehicle counts, error in the street reference data used for geocoding, and error introduced by the geocoding process.

The positional error of street reference data is closely related to the scale of the data. For example, data at a scale of 1:24,000 will be accurate to within 12 m 90% of the time based on National Map Accuracy Standards (NMAS) (U.S. Geological Survey 1999). The widely used Topologically Integrated Geographic Encoding and Referencing (TIGER) street data from the U.S. Census Bureau meets the standard for 1:100,000 scale maps and will be accurate to within 50 m 90% of the time, although the most recent versions of TIGER data are expected to be of greater accuracy (U.S. Census Bureau 2000).

These errors are potentially additive, presenting a major challenge to fine-scale analysis which relies on small positional error. Of the several types of errors listed above, only the positional error of the major roads has received attention in the literature on the effects of traffic-related air pollution on children (Ong et al. 2006; Wu et al. 2005). Both these studies determined the reliability of using moderately accurate street reference data for geocoding by manually realigning it with higher quality reference data. Geocoding results were found to be very unreliable for analysis using short distances.

The main objective of our study was to determine the influence of the quality of geocoding on the analysis of the effect of traffic-related air pollution on children at school locations. Two aspects of geocoding quality are considered: positional accuracy (difference between gecoded locations and the actual school locations) and repeatability (difference between the results of different geocoding techniques). We selected school locations for this study because they represent the second most important location where children spend their time, after the home residence, and because geocoding has been widely employed in studies that have tried to determine exposure potential to air pollution at school locations (e.g., Chakraborty 2001; Green et al. 2004; Ong et al. 2006). We selected multiple geocoding techniques to determine the sensitivity of the results to variability in geocoding quality. The first technique consists of using county street centerlines. This represents the highest quality street reference network available for free in most areas. The second technique consists of TIGER roads from the U.S. Census Bureau (2000). Although its positional and attribute accuracy is often inferior to that of other data sources, it is a very widely used data source for geocoding because it is free and covers all of the United States. The third and fourth techniques consist of using the services of two commercial geocoding firms, a common practice among environmental exposure researchers who need to geocode addresses.

## Methods

The study design relies on a comparison between the results of geocoding and the actual school locations. We obtained addresses for all public schools in Orange County, Florida, for 2005 from the Orange County School Board (2005). We determined the actual location of each school by using a detailed 1:2,000 digital parcel database for 2005 for Orange County (Orange County Property Appraiser 2005). We identified all 153 schools in this database through manual searches based on the fields for physical address and ownership (i.e., Orange County School Board). After we identified the correct property, we overlaid the parcel boundaries on digital 1-m color orthophotography for 2005 in ArcGIS 9 (ESRI Inc., Redlands, CA). In this overlay the parcel boundaries are shown and the aligned imagery provides a detailed look at building(s) at the school site. We used this overlay to manually digitize the exact outline of the school building(s). We then created a single centroid from the digitized building(s) for each school to represent the “true” location.

We geocoded the schools using a 1:5,000 street centerline network from Orange County for 2005 (Orange County Growth Management 2005) and the TIGER 2000 streets from the U.S. Census Bureau (2000), both using ArcGIS 9. Identical settings were employed, including address locator style, field types used in the address locator, minimum match score, and spelling sensitivity. For any records that did not automatically produce a reliable match, exhaustive manual interactive matching was carried out to achieve the highest possible number of reliable matches. We used a perpendicular offset of 10 m in the placement of the geocoded locations, based on the typical width of the right-of-way of local roads of 15–20 m. We also sent out the address file for processing to two commercial geocoding firms, which we refer to as Firm A and Firm B. It was not possible to specify geocoding settings, but we used the final match codes to identify the high-quality matches and used only those in further analysis. Match rates for the four techniques were 94.8% for street centerlines, 89.5% for TIGER roads, 90.2% for Firm A, and 90.9% for Firm B. For the remainder of the analysis, we used only records that could reliably be geocoded using all four techniques (*n* = 126).

We determined the positional accuracy of the geocoded locations by measuring the straight-line distance between the geocoded location and the actual location of the particular school. This was repeated for each of the four sets of geocoded results. We characterized error distributions using descriptive statistics and cumulative distribution functions.

We determined exposure potential to traffic-related air pollution using proximity to high traffic intensity roads. We obtained a detailed 1:24,000 road network for the State of Florida from the Florida Department of Transportation (FDOT 2005) with average annual daily traffic (AADT) values for 2005 for each road segment. We selected road segments with an AADT of ≥ 25,000 for further analysis. For each school we determined the straight-line distance to the nearest road segment using ArcGIS 9 for the actual location as well as for the four geocoded locations. We also created straight-line buffer zones around the road segments with an AADT of 25,000 as discrete representations of distances commonly used in studies on traffic-related air pollution. We used buffer radii of 50, 100, 150, 250, 500, and 1,000 m.

**Figure 1:**
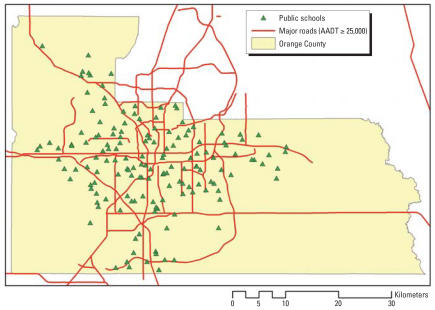
Locations of schools and major road network in Orange County, Florida (major roads with AADT ≥ 25,000 vehicles per day).

Figure 1 shows the actual locations of the schools in Orange County as well as the major road network with AADT values of ≥ 25,000 vehicles per day.

We determined bias and error introduced by geocoding by comparing the results of proximity analysis using the actual school locations and the fours sets of geocoding results. Specifically, we determined the number of correctly and incorrectly classified schools using geocoding for the buffer zones described above. This required determining for each buffer zone which schools are actually located within that distance, which schools are correctly classified as being located within that distance using geocoding (confirmed positives), which schools are incorrectly classified as being located outside that distance (false negatives), which schools are incorrectly classified as being located within that distance (false positives), and which schools are correctly classified as being located outside that distance (confirmed negatives). We determined the overall agreement between the results for actual school locations and geocoded locations for each distance using percentage false negatives, percentage false positives, sensitivity, and specificity. We repeated this analysis for each of the four sets of geocoded locations.

## Results

**Figure 2:**
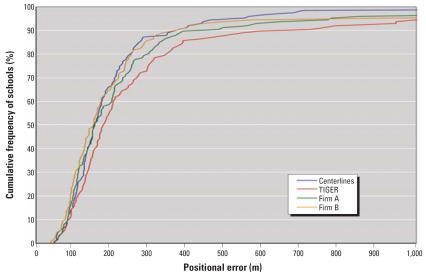
Cumulative distribution functions of positional error in geocoded locations of schools in Orange County, Florida (*n* = 126).

Figure 2 shows the cumulative distribution functions of the positional error in the geocoding results, and Table 1 provides descriptive statistics. The first characteristic to notice is the non-normal distribution of errors; the mean is much higher than the median in all four distributions, and the distributions are highly skewed due to the occurrence of a small number of very large error values.

The distributions are relatively similar for the four techniques considered, but the error is consistently larger for the TIGER results. For example, the 50th percentile is 155 m for street centerlines, 178 m for TIGER roads, 153 m for Firm A, and 151 m for Firm B. At higher percentiles, the curves are a bit further apart, suggesting that street centerline geocoding is the most accurate technique. For example, the 95th percentiles are 227 m for street centerlines, 302 m for TIGER roads, 255 m for Firm A, and 237 m for Firm B. To characterize the overall distribution the 95% root mean square error (RMSE) is a robust statistic for non-normally distributed positional errors; the 95% RMSE is 196 m for street centerlines, 306 m for TIGER roads, 235 m for Firm A, and 210 m for Firm B. These results strongly suggest that the positional error in school locations for all types of geocoding is quite large, and that the error is much higher when using TIGER roads.

**Figure 3:**
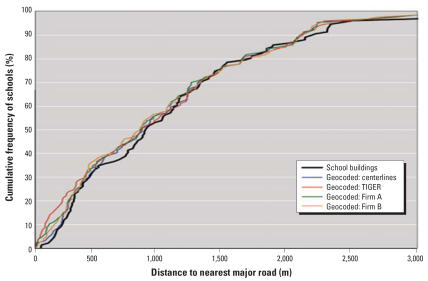
Cumulative distribution functions of distance to major roads of school locations in Orange County, Florida (*n* = 126) mapped using five different techniques.

Figure 3 shows the cumulative distribution functions of the distance to the nearest major road for the actual school locations and the four sets of geocoded locations. These distributions can be used to examine bias. “Bias” is defined here as a consistent over- or underestimation of the number of schools at risk. If there were no bias, the curves would be nearly identical, and any consistent difference would indicate over- or underestimation. Figure 3 reveals that the curves for the four geocoding techniques are consistently higher than the curve for the actual school locations at most distance values < 1,000 m. This indicates that the geocoding results provide a consistent overestimation of the potential schools at risk. Comparing the results for the four sets of geocoded locations reveals that the use of TIGER roads results in the largest bias at distances of up to 500 m.

Although the results in Figure 3 reveal the bias introduced by geocoding, they do not show the occurrence of error in the form of false positives and negatives. Therefore, for every buffer radius considered, we determined the number of schools within that distance using actual locations and the geocoded locations, as well as the agreement between the results in terms of correctly classifying schools. Table 2 shows the results of this analysis.

Table 2 shows the number of schools located within each buffer radius based on actual and geocoded locations. For each of the four sets of geocoded locations and for nearly all distances considered, the number of schools at risk is consistently higher using geocoded locations than using actual locations, confirming the strong bias toward an overestimation of the number of schools at risk as already shown in Figure 3. Table 2 also shows the confirmed positives, false negatives, false positives, and confirmed negatives. The percentage of false negatives and positives is calculated relative to the total sample size (*n* = 126). The percentage false of positives is consistently higher than the percentage of false negatives, confirming the strong bias toward an overestimation of the number of schools at risk.

Although the number of false positives and negatives as a percentage of the total sample is relatively small, at short distances the error in classification is very substantial. For example, three schools are actually located within 100 m of a major road, and street centerline geocoding correctly identifies only one of them. Centerline geocoding identifies four other schools within 100 m, but these are all false positives. This error is more formally expressed in the measure for sensitivity, which is the percentage of schools located within a specific buffer radius that were correctly identified using geocoding. As can be seen in Table 2, the values for sensitivity are very low at short distances and gradually increase to values of 90% or higher at distances of 500–1,000 m. The very low values for sensitivity at short distances strongly indicate that the results at these distances are very unreliable. A final measure of agreement is the specificity, which is the percentage of schools located outside a specified buffer radius that are correctly identified using geocoding. Values are consistently high, with most values > 90% at all distances considered. This is largely owing to that fact that although the identification of schools at risk is very unreliable at short distances, their prevalence is quite low.

When comparing the results of the four geocoding techniques, the results using TIGER roads are the least reliable, with the highest counts of false positives and the lowest values for sensitivity and specificity. Results from Firm A are the second least reliable, with the second highest counts of false positives and the second lowest values for sensitivity and specificity. Results for street centerlines and commercial Firm A are very similar, and a determination of which method is most reliable varies with the exact distance value and parameter chosen.

The results in Table 2 strongly suggest that the identification of schools at risk based on proximity is unreliable at short distances. This raises an important question: At what distance, if any, the results do become reliable? We use the measure for sensitivity to try to answer this. If a value of 90% for sensitivity is deemed acceptable, the minimum distance needed to achieve reliable results is 500 m for street centerlines, TIGER, and Firm B, and 1,000 m for Firm A. If a value of 95% is used, the minimum distance is 500 m for street centerlines and Firm B, and 1,000 m for TIGER and Firm A. These results suggest that the reliability of proximity-based identification of schools at risk varies with geocoding quality, but that overall the results are not reliable at distances < 500 m.

## Discussion

The positional error in geocoded locations of schools was very high relative to the accuracy requirements for fine-scale proximity analysis; a median error of 155 m for street centerline geocoding, 178 m for TIGER roads, 153 m for Firm A, and 151 m for Firm B, and 95% RMSE values of 196, 306, 235 and 210 m, respectively. These estimates are substantially higher than those found in previous studies (Bonner et al. 2003; Cayo and Talbot 2003; Dearwent et al. 2001), which have looked mostly at residential addresses. The larger positional errors for schools are a result of the much larger parcels on which most schools are located, relative to residential properties. Both larger parcel sizes and variability in parcel size along a street segment can contribute to larger positional errors.

The amount of bias and error introduced by the positional error in geocoding is substantial. As a general rule, spatial data must be much more accurate than the minimum distance used in spatial analysis for the results to be meaningful (Diggle 1993; Waller 1996); this rule is clearly not met when using the results of geocoding of schools in fine-scale analysis in the order of 100 or even several hundred meters.

**Figure 4:**
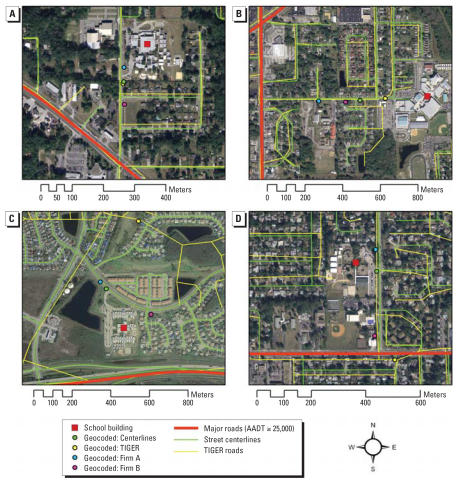
Examples of positional error in geocoding of school locations in Orange County, Florida. (*A*) Effect of driveway. (*B*) Misplacement along street segment. (*C*) Effect of parcel size. (*D*) Combined effects.

Figure 4 shows the geocoding results for selected areas that we use to discuss several common scenarios. In each of the four scenarios the school building centroid and the geocoded locations are shown, in addition to the street centerlines, TIGER road, and the major roadways (AADT ≥ 25,000). Street reference networks from the commercial firms are not available, but the placement of the geocoded locations suggests they are relatively similar to the street centerlines.

The first general observation from Figure 4 is the influence of the positional accuracy of the street network. The street centerlines are almost perfectly aligned with the aerial imagery, suggesting a very high positional accuracy. This suggests that the positional error in geocoding using this reference network is largely attributed to the errors in the placement of the geocoded location along the street segments, not the position of the network itself. The positional error in the TIGER roads, however, is substantial, and misalignment with the imagery is observed for almost every segment. Errors up to 50 m are very common, and errors of several hundred meters also occur. This explain the much larger positional error found in the geocoding results using TIGER roads.

A related observation from Figure 4 is the influence of the positional accuracy of the major road network. In most of the study area the major road network is very well aligned with the imagery and is a near-perfect match with the street centerlines. This suggests that, except for TIGER roads, misalignment of spatial data sets is not a significant factor, as it was in previous studies (Ong et al. 2006; Wu et al. 2005). Also, very few schools are located directly on major roads; most are located on side streets of major roads, making the placement along the side street relative to the major roads the critical factor in correctly identifying schools at risk.

Figure 4A shows a typical scenario where the geocoded locations are in relatively close proximity to each other, but at some distance from the actual school. There are several factors at work here. First, there is the “driveway” effect: Many schools are located on a fairly large parcel and a private driveway leads from the road to the actual building. This driveway does not appear in street network data. So even if a geocoded location were directly in front of the school parcel, it would be at some distance from the actual building. Second, there is misplacement along the street network, as evidenced in particular for the result for Firm B in Figure 4A. Geocoding relies on linear interpolation of the actual address within the address range for the street segment. If the address range for the segment does not reflect the true addresses, or the parcels along the segment are not uniform, locations are misplaced along the segment relative to the actual location. As a result of these two effects, all geocoded locations in Figure 4A are placed closer to the major road than the actual school building, resulting in a (potential) false positive. This particular scenario accounts for most false positives in our study.

Figure 4B shows a scenario similar to 4A, but in this case the geocoded locations are further away from the major road than the actual school location, resulting in a (potential) false negative. This particular scenario accounts for most false negatives in our study. Figure 4B also illustrates the substantial error in the TIGER roads, which in this case translates in only a minor additional error in the geocoding results.

Figure 4C illustrates a scenario where the school is located on a very large parcel, resulting in substantial driveway effect. In this case the actual school buildings are much closer to the major roadway, producing a (potential) false negative. This scenario illustrates that even the most accurate geocoding result (i.e., a location mapped directly in front of or inside the school parcel) does not capture the school location sufficiently accurate for fine-scale proximity analysis. The scenario again illustrates the substantial error in the TIGER results.

Figure 4D illustrates a scenario where the driveway effect is limited, but the placement of the geocoded locations along the segment varies substantially among the four techniques. Results for street centerlines and Firm B are quite accurate, resulting in a correct classification of whether the school is at risk. Results for TIGER and Firm A are placed at several hundred meters from the actual school location, resulting in incorrect classification.

When comparing the four different sets of geocoded locations in Figure 4, some relevant patterns emerge. First, results for street centerlines and Firm B are consistently close to each other and typically the most accurate relative to the actual school location. This suggests a high degree of repeatability of geocoding when considering only these two techniques. Results for Firm A are typically at some distance from the previous two locations along the same street segment, and typically at a larger distance from the actual location. Results for TIGER show much more variability, with several locations being placed incorrectly due to both positional and attribute errors in the TIGER roads. These examples illustrate the general findings for positional errors in the geocoding results and the reliability of the proximity-based exposure analysis: Street centerlines and Firm B are most accurate and reliable, followed by Firm A, with TIGER a distant fourth.

One final pattern that emerges from the four scenarios is that most of the error in the classification of schools results from errors in the placement of the geocoded locations along the street segment, not from the error in the distance from the road. This is clearly illustrated in Figures 4A–C, where placing the geocoded locations at the same distance from the road as the actual school location would not improve the correct identification of schools at risk. This is a result of the fact that in our study area very few schools are located directly on a major road with high traffic densities but instead on side streets perpendicular to major roads.

The four scenarios presented in Figure 4 all contribute to errors in the proximity analysis in the form of false positives and negatives. If these errors were strictly random, the number of false positives and negatives would be the same. However, we observed a consistent bias in the results of the proximity analysis with many more false positives than false negatives. The scenarios in Figures 4A and 4D contribute to false positives, and an inspection of the entire data set reveals that these are in fact the most common scenarios for false positives. The observed bias in the proximity analysis, therefore, is a result of a combination of positional and attribute errors in the street reference data, limitations of the linear interpolation algorithm used in geocoding, and the inherent limitations of a street network to capture the actual locations of school buildings located on large parcels.

When considering the results for the four sets of geocoded locations, several observations regarding the accuracy and repeatability of geocoding emerge. First, the results from the TIGER 2000 roads were by far the least accurate, and the use of these data for fine-scale spatial analysis should be discouraged despite their widespread availability and low cost. When updated versions of the TIGER data become available for a particular area, they should be checked to determine whether they provide improved accuracy and reliability. Second, the differences between the results from the two commercial geocoding firms suggest a substantial lack of repeatability, which is not reflected in the cost (Firm B was more accurate and cheaper). Commercial geocoding is available for most areas at relatively low cost, but results typically do not include a measure of positional accuracy, and their use for fine-scale analysis needs to be carefully considered. Third, the use of street centerlines produced the most accurate and reliable results (and results for Firm B were very similar), but this type of data may not be available everywhere, and their quality may vary between jurisdictions, limiting usefulness for larger data sets across multiple jurisdictions.

One very important limitation of this study is that school locations are somewhat unusual in that they are typically located on large parcels relative to other types of locations (such a private residences). As a result, even a geocoded location very close to or inside the correct parcel may be at a substantial distance from the actual school building(s). The presence of large parcels along a street segment also causes errors in the linear interpolation used in the placement of geocoded locations along the street segment. In addition, many schools in suburban areas are off-set from the street with a private driveway. Combined, these factors result in large positional errors in geocoded locations. These errors are typically larger than for other types of locations. For example, Cayo and Talbot (2003) determined the positional error of private residences using the same technique (distance between geocoded locations and building centroid) across an urban–rural gradient and found a median positional error of 38 m for urban areas, 78 m for suburban areas, and 201 m for rural areas. Values for the 90th percentile were 96, 306 and 1,544 m, respectively. The schools in our study are located mostly in suburban areas, and results for the most accurate geocoding using street centerlines produced a mean of 155 m and a 90th percentile of 211 m. The results for the school locations in our study are substantially less accurate than private residences in typical urban and suburban areas, but more accurate than those in rural areas. Based on these error values, therefore, the observed bias and error in the proximity-based exposure analysis is likely to be less for private residences in urban areas, but still substantial at short distances of 100 or possibly several hundred meters. The results for other types of locations, however—such as hospitals, shopping malls, apartment complexes, businesses, office parks—are more likely to be similar to those found in our study for schools because they share the same characteristics of large parcels and private driveways or access roads.

A second limitation is that our study employed only a relatively small sample of schools for a single county. As a result, our findings are limited to similar areas where schools are located primarily in suburban areas on relatively large parcels. Positional errors in geocoding in higher-density urban areas are likely to be smaller, with corresponding lower bias and error in proximity-based analysis of environmental exposure. A related limitation is the fact that the study area is located in the United States and that the geocoding results therefore are typical for the street reference data commonly available in the United States. Results in other jurisdictions may be more reliable if more accurate geocoding techniques are available.

## Conclusions

Positional errors in geocoding of school locations introduce substantial bias and error in the analysis of the effects of traffic-related air pollution on children. Alternatives to street geocoding need to be considered, including parcel-based geocoding, address point geocoding, the use of ortho-imagery, and field observations using global positioning systems. Digital parcel data in GIS format and high resolution ortho-imagery are becoming more widely available, although coverage might be spotty across a large study area of multiple jurisdictions. The skill level required to use these types of data in GIS is not much higher than that needed for street geocoding, and the data are mostly available for free. However, the effort required to compile and process these detailed data is substantial, and presents a major barrier to more widespread adoption. For very large data sets, including those covering more than a few individual counties, street geocoding is likely to remain a more cost-effective solution.

The widespread availability of powerful geocoding tools in commercial GIS software and the interest in spatial analysis at the individual level have made address geocoding a widely employed technique in epidemiologic studies. Although some of the limitations of geocoding have been addressed in recent review articles in public health and epidemiology journals (McElroy et al. 2003; Rushton et al. 2006), most studies have employed geocoding without much consideration of its inherent limitations. Match rates have received most recognition, and the positional error has been assumed to be small in magnitude and random in its effect on analysis results. We have shown in this study that the positional error in geocoding is neither small nor random, and that caution in the use of geocoding results for epidemiologic studies is warranted. TIGER data in particular are prone to very large errors.

Limitations of this study include the unique nature of school locations, which results in larger errors than are typically encountered for residential locations, and the fact that only a single county in the United States was considered. Despite these limitations, the study provides insights into the nature of the errors that can be expected for other types of locations and jurisdictions.

Geocoding is very appealing as a data processing step because it provides a high degree of automation, but the results are not accompanied by reliability estimates for its quality other than match scores. The use of street reference data of high positional accuracy and currency is also no guarantee the positional accuracy of geocoding will be sufficient, and the use of alternatives should be considered when fine-scale analysis is required.

## Figures and Tables

**Table 1:** Summary statistics for the positional error (in meters) of geocoded locations of schools (*n* = 126) in Orange County, Florida, using four different techniques.

Statistics	Centerlines	TIGER	Firm A	Firm B
Mean	219	351	300	461
Median	155	178	153	151
Standard deviation	272	604	602	2,330
Minimum	50	49	48	39
Maximum	2,302	4,379	5,565	2,596
90th percentile	211	271	238	218
95th percentile	227	302	255	237
95% RMSE^a^	196	306	235	210

a 95% RMSE is the root mean square error of the error distribution after removing 5% outliers. It is more common to use the 100% RMSE, but for non-normally distributed data the removal of 5% outliers before determining the RMSE value produces a more robust accuracy statistic.

**Table 2:** Bias and error in determining schools (*n* = 126) at risk based on proximity to major roads in Orange County, Florida.

	No. of schools within buffer zone						Measures of agreement				
Geocoding type, buffer radius (m)	School buildings	Street geocoding	Confirmed positives	False negatives	False positives	Confirmed negatives	Prevalance (%)^a^	False negatives (%)^b^	False positives (%)^c^	Sensitivity (%)^d^	Specificity (%)^e^
Street centerlines											
50	1	3	0	1	3	122	0.79	0.79	2.38	0.00	97.60
100	3	5	1	2	4	119	2.38	1.59	3.17	33.33	96.75
150	6	9	4	2	5	115	4.76	1.59	3.97	66.67	95.83
250	17	20	12	5	8	101	13.49	3.97	6.35	70.59	92.66
500	44	44	42	2	2	80	34.92	1.59	1.59	95.45	97.56
1,000	69	71	66	3	5	52	54.76	2.38	3.97	95.65	91.23
TIGER roads											
50	1	9	0	1	9	116	0.79	0.79	7.14	0.00	92.80
100	3	15	1	2	14	109	2.38	1.59	11.11	33.33	88.62
150	6	20	4	2	16	104	4.76	1.59	12.70	66.67	86.67
250	17	29	13	4	16	93	13.49	3.17	12.70	76.47	85.32
500	44	46	40	4	6	76	34.92	3.17	4.76	90.91	92.68
1,000	69	70	67	2	3	54	54.76	1.59	2.38	97.10	94.74
Commercial Firm A											
50	1	5	0	1	5	120	0.79	0.79	3.97	0.00	96.00
100	3	11	1	2	10	113	2.38	1.59	7.94	33.33	91.87
150	6	14	4	2	10	110	4.76	1.59	7.94	66.67	91.67
250	17	23	11	6	12	97	13.49	4.76	9.52	64.71	88.99
500	44	46	39	5	7	75	34.92	3.97	5.56	88.64	91.46
1,000	69	72	66	3	6	51	54.76	2.38	4.76	95.65	89.47
Commercial Firm B											
50	1	3	0	1	3	122	0.79	0.79	2.38	0.00	97.60
100	3	8	2	1	6	117	2.38	0.79	4.76	66.67	95.12
150	6	12	5	1	7	113	4.76	0.79	5.56	83.33	94.17
250	17	22	10	7	12	97	13.49	5.56	9.52	58.82	88.99
500	44	48	43	1	5	77	34.92	0.79	3.97	97.73	93.90
1,000	69	72	67	2	5	52	54.76	1.59	3.97	97.10	91.23

a Number of schools residing within the buffer radius as a percentage of all schools within the study area (*n* = 126).

b Number of false negatives as a percentage of all schools within the study area (*n* = 126).

c Number of false positives as a percentage of all schools within the study area (*n* = 126).

d Number of confirmed positives as a percentage of all schools within the study area (*n* = 126).

e Number of confirmed negatives as a percentage of all schools within the study area (*n* = 126).
